# EEG-based emotion recognition using a temporal-difference minimizing neural network

**DOI:** 10.1007/s11571-023-10004-w

**Published:** 2023-09-11

**Authors:** Xiangyu Ju, Ming Li, Wenli Tian, Dewen Hu

**Affiliations:** College of Intelligence Science and Technology, National University of Defense Technology, Changsha, China

**Keywords:** EEG, Emotion recognition, Maximum mean discrepancy, Temporal-difference minimizing neural network

## Abstract

Electroencephalogram (EEG) emotion recognition plays an important role in human–computer interaction. An increasing number of algorithms for emotion recognition have been proposed recently. However, it is still challenging to make efficient use of emotional activity knowledge. In this paper, based on prior knowledge that emotion varies slowly across time, we propose a temporal-difference minimizing neural network (TDMNN) for EEG emotion recognition. We use maximum mean discrepancy (MMD) technology to evaluate the difference in EEG features across time and minimize the difference by a multibranch convolutional recurrent network. State-of-the-art performances are achieved using the proposed method on the SEED, SEED-IV, DEAP and DREAMER datasets, demonstrating the effectiveness of including prior knowledge in EEG emotion recognition.

## Introduction

Emotion is a complex, subjective expression of human beings and is often accompanied by external manifestations such as behavioural and physiological responses (Picard et al. 2001). As one of the most significant topics in emotion research, emotion recognition with physiological signals plays an important role in human–computer interaction (Fiorini et al. 2020). Emotion recognition enables machines to perceive human mental states and greatly enhances machine learning and prediction (Cowie et al. 2001). Previous works have shown that electroencephalography (EEG) contains much information about emotion, making it possible to decode emotions based on EEG signals (Pan et al. 2016; Liu et al. 2017; Goshvarpour and Goshvarpour 2019; Moon et al. 2020). In addition, EEG has the advantages of portability, low cost, and high time resolution and reflects subtle emotional changes, so it has been widely used in emotion recognition (Al-Nafjan et al. 2017; Alarcão and Fonseca 2019; Torres et al. 2020; Huang et al. 2021).

Emotion recognition methods can be roughly divided into shallow models and deep models (Islam et al. 2021). In these models, feature extraction and processing are important steps. In shallow models, the features of raw EEG signals are extracted and manually selected. For example, Duan et al. used the differential entropy (DE) of each EEG channel as a discriminative feature of emotion and shallow classifiers such as support vector machine (SVM) and K-nearest neighbour (KNN) to achieve emotion recognition (Duan et al. 2013). Mohammadi et al. used wavelet transforms to extract features from the time–frequency domain and detected emotional states using SVM and KNN (Mohammadi et al. 2017). Li et al. adopted 9 time–frequency domain features, such as the Hjorth parameter (HP), and 9 nonlinear dynamic system features, such as spectral entropy (SE), and explored the importance of different EEG features in emotion recognition (Li et al. 2018a, b). Because the features in shallow models are manually designed based on neuroscience knowledge, they are quite discriminative with respect to emotion recognition. However, because classifiers are usually simple, the manually designed features are hard to further analyse and fully use in shallow models.

Different from shallow models, deep models use complex neural networks to achieve both feature extraction and emotion classification. Because of the complexity of deep networks, deep models can make full use of the extracted features, and high classification accuracy can be achieved. For example, Cimtay et al. used a convolutional neural network (CNN) to extract the spatial features of emotional activities, and accuracies of 86.56% and 72.81% were achieved on the SEED and DEAP datasets, respectively (Cimtay and Ekmekcioglu 2020). Wang et al. extended the dimension of CNN and designed EmotioNet to obtain spatial features (Wang et al. 2018). Alhagry et al. used long short-term memory (LSTM) to represent temporal features from EEG signals and obtained a classification accuracy of 85.65% for arousal and 85.45% for valence on the DEAP dataset (Alhagry et al. 2017). Ma et al. proposed a multimodal residual LSTM (MMResLSTM) network containing temporal shortcut paths to extract temporal representations (Ma et al. 2019). Yang et al. proposed hybrid neural networks that combined a CNN and a recurrent neural network (RNN) to learn both spatial and temporal representations of EEG signals and achieved high performance, with mean accuracies of 90.80% and 91.03% for valence and arousal emotion classification, respectively (Yang et al. 2018). Tao et al. proposed an attention-based convolutional recurrent neural network (ACRNN) to extract spatial and temporal attentive features from EEG signals and achieved average accuracies of 93.72% and 93.38% for valence and arousal classification on the DEAP dataset (Tao et al. 2020).

However, to extract discriminative features, deep models usually require a large amount of data(He et al. 2021). If the amount of data is insufficient, the features automatically extracted by deep models are not as discriminative as manually designed features, which are based on neuroscience knowledge. Therefore, some researchers have attempted to combine manually designed features in shallow models and deep networks in deep models to identify emotions more accurately. In these methods, the manually designed features are extracted first (initial features), and then from the initial features, more discriminative features are further extracted by deep neural networks (advanced features).

Because such methods combine the advantages of shallow models and deep models in feature extraction and selection, they have gained increasing interest (Du et al. 2022; Feng et al. 2022). For instance, Li et al. used a two-dimensional CNN to further extract manually selected DE features from different channels (Li et al. 2018a, b). Song et al. proposed a dynamic graph convolutional neural network (DGCNN) to extract more discriminative features from 5 initial features, such as the DE and the power spectral density (PSD) (Song et al. 2018). Zhong et al. proposed a regularized graph neural network (RGNN) to capture spatial relations based on the EEG adjacency matrix (Zhong et al. 2022). To extract the temporal advanced representations of initial features, LSTM networks, recurrent neural networks (RNNs) and similar tools as well as convolutional networks are generally used (Algarni et al. 2022; Zhu et al. 2022). Yin et al. designed the emotion classification LSTM and GCNN (ECLGCNN), integrating GCNN and LSTM to further extract both temporal and spatial characteristics from DE features (Yin et al. 2021). Chen et al. proposed both cascaded and parallel hybrid convolution recurrent neural networks to learn the spatial and temporal high-level discriminative information from 2D PSD mesh sequences and achieved over 93% for valence and arousal on the DEAP dataset (Chen et al. 2020). Shen et al. extracted advanced spatiotemporal patterns from the initial features using a four-dimensional convolutional recurrent neural network (4D-CRNN) (Shen et al. 2020). Xiao et al. proposed an attention-based neural network, which fused information on different domains and captured discriminative patterns by adding attention mechanisms, and achieved the highest accuracy of 96.25% on the SEED dataset based on the work of Shen (Xiao et al. 2022).

However, when using the above methods, it is still challenging to make efficient use of the knowledge of emotional activities when extracting advanced features from initial features. In fact, EEG patterns of emotional activities are relatively stable over time (Li et al. 2019; Zheng et al. 2019a, b). It was reported that emotion states in brain signalling tend to last for about 5–15 s before transitioning to another state (Kragel et al. 2022). Some other researchers reported that the brief duration of an emotion lasts for approximately 1–10 s (Ekman 1992; Levenson 2003). And in many emotional or physiological researches, to confirm the theoretical perspectives on the duration and temporal characteristics of emotional responses, the time window limited to 10 s was widely chosen (Mauss et al. 2005; Sze et al. 2010; Dan-Glauser and Gross 2013; Lohani et al. 2018). On the popular emotion datasets such as SEED, DEAP and DREAMER, the subjects are continuously stimulated for a long period, during which their emotion is relatively stable. In this paper, we attempt to design a deep neural network inspired by the time stability of emotional activity. The maximum mean discrepancy (MMD) technology is used to evaluate the difference in EEG features across time, and the MMD value is minimized by a multibranch convolutional recurrent network during emotion recognition. The proposed method is referred to as the temporal-difference minimizing neural network (TDMNN).

The innovation and contribution of this work can be outlined as follows: a) Based on the prior knowledge that emotion varies slowly across time, we introduce the temporal stability into the EEG emotion recognition. b) By employing the MMD and designing a feature scrambling module to effectively evaluate the variation of EEG features across time. c) The experimental results demonstrate that TDMNN can achieve state-of-the-art performance on benchmark datasets. This indicates that including prior knowledge of the time stability of emotional activity is helpful to achieve efficient emotion recognition.

The rest of this paper is organized as follows: We describe our proposed method in the Method section. In the Experiment section, the datasets, experiment setting, results and discussion are presented. Finally, conclusions are given in the Conclusion section.

## Method

**Fig. 1:**
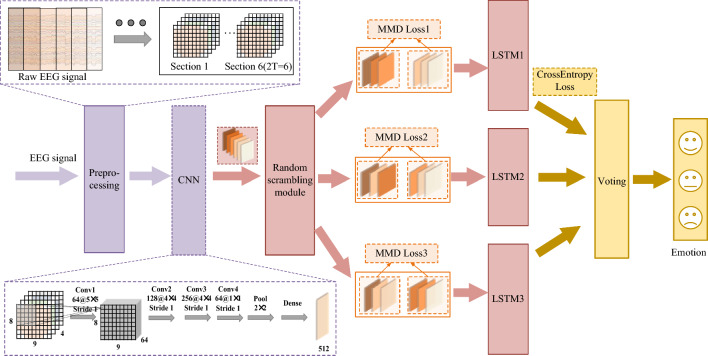
An overview of the proposed TDMNN. It consists of input conversion, a convolutional recurrent network with a multibranch temporal-difference evaluation module and a voting classifier. The convolutional recurrent network can be divided into the CNN, the temporal-difference evaluation module and the parallel LSTM

In this paper, a temporal-difference minimizing neural network is designed for EEG-based emotion recognition, as illustrated in Fig. 1. It consists of the preprocessing, convolutional recurrent network with a multibranch module, and voting classifier. First, in preprocessing, the spectral, spatial and temporal features of the EEG signal are integrated into the four-dimensional feature representation (Shen et al. 2020). Then, a convolutional recurrent network with a multibranch module is designed to further extract the features and evaluate the temporal stability of emotional activities. Finally, the emotion classification result is predicted by a voting classifier. In the remainder of this section, we will introduce the network architecture proposed in this paper.

### Preprocessing

**Fig. 2:**
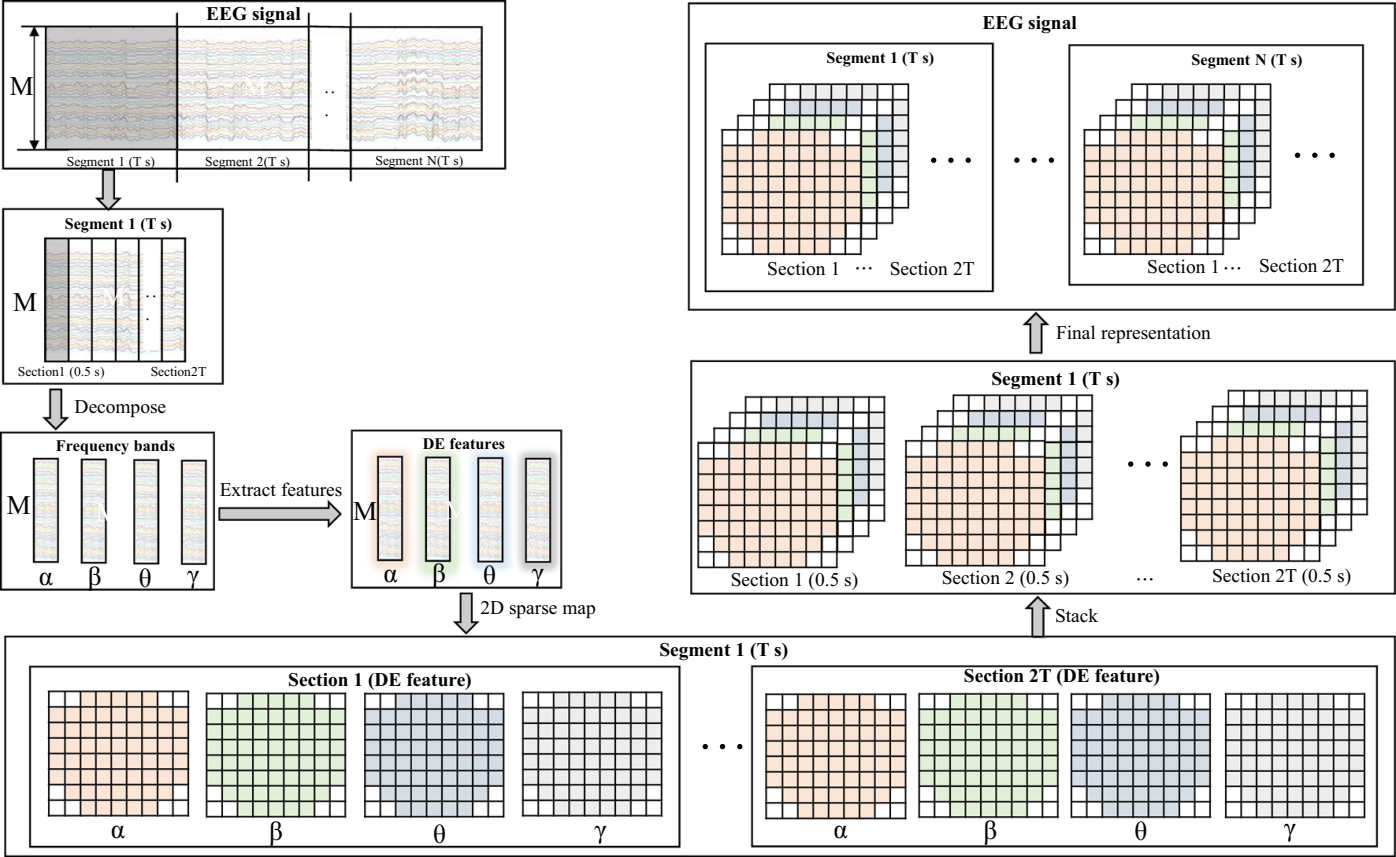
The input conversion of EEG signals. The raw EEG signals can be divided into $N$ segments. For each $T$ s EEG signal segment, the EEG signal is transformed into a 4D representation that is divided into a two-dimensional spatial EEG arrangement, four parallel frequency bands, and $N$ segments with $T$ s length

The input conversion is depicted in Fig. 2. As in previous works (Xiao et al. 2022), the raw EEG signals are converted into four-dimensional differential entropy (DE) feature segments. The four dimensions consist of 2 spatial dimensions, spectral dimension and temporal dimension.

The details are as follows. First, we split raw EEG signals into *N* segments with no overlapping *T*s lengths, and each segment can be divided into 2* T* continuous sections (Yang et al. 2018). We set the length of EEG segment (parameter *T*) as 3. Then, each section is decomposed into 4 frequency bands (θ [4 ~ 8 Hz], α [8 ~ 14 Hz], β [14 ~ 31 Hz], and γ [31 ~ 51 Hz]). Next, the DE features from each band are extracted and arranged onto a compact 2D map (Shen et al. 2020). For a fixed length EEG segment, DE is equivalent to the logarithm energy spectrum in a certain frequency band (Zheng and Lu 2015). It is estimated using Shi’s algorithm (Shi et al. 2013), which has balance ability of discriminating EEG pattern between low and high frequency energy. Finally, the 2D maps of 4 frequency bands are stacked to obtain the 3D features. Through the above operations, the raw EEG signals are converted into *N* segments, denoted as $S_{N}$, $S_{N} \in R^{9 \times 8 \times 4 \times 2T}$.

### Convolutional recurrent network with a multibranch temporal-difference evaluation module

With the prior knowledge that emotion varies slowly across time, a convolutional recurrent network with a multibranch temporal-difference evaluation module is designed to extract advanced spatiotemporal features from initial features (four-dimensional DE features).

We extract the discriminative spatial features by using the CNN, which is the same as the 4D-CRNN model proposed by Shen et al. (2020), as shown in Fig. 1. And segment $S_{N}$ is converted into $Pt$, $Pt \in R^{512 \times 2T}$, which is the spatial feature of the DE feature. According to our assumption on emotion stability across time, the features of the emotion activity vary slightly during a period (*T* seconds). In our work, the period contains 2* T* feature vectors, which can be divided into 2 groups (each contains *T* vectors). The stability can be measured by the distribution difference between the two groups. The maximum mean difference (MMD) is a statistical measure to quantify the discrepancy between two probability distributions. It is easy to implement, memory-efficient and widely used in the domain adaption to narrow the discrepancy between the source domain and the target domain (Chen et al. 2021). Thus, in our study, we used MMD to evaluate the difference of EEG emotional features at different time during a period. The MMD is defined in Eq. (1), where the distribution of $x$ is $p$ and that of $y$ is $q$. Because $E$ cannot be calculated directly, we can use the mean value instead of $E$, as shown in Eq. (2). There are $n$ samples of $X$ and $m$ samples of $Y$.

1 $${\text{MMD}}\,\left( {F,p,\,q} \right) = \mathop {\sup }\limits_{{\left\| f \right\|_{\rm H} \le 1}} E_{p} [f(x)] - E_{q} [f(y)]$$

2 $${\text{MMD}}\,\left( {F,p,q} \right) = \left\| \frac{1}{n} \right.\sum\limits_{i = 1}^{n} {f(x_{i} } ) - \left. {\frac{1}{m}\sum\limits_{j = 1}^{m} {f(y_{j} } )} \right\|_{H}$$

We design a random scrambling module to effectively evaluate the variation of EEG features across time. 2 T feature vectors in $Pt$ are randomly scrambled 3 times and then divided into 2 groups by the module. In the process of training, the MMD value between the groups is minimized as the loss to narrow the difference in EEG features before and after time. Therefore, the temporal-difference evaluation module can make full use of the difference among the continuous sections to obtain the time stability of emotional activities. To make efficient use of the temporal difference of EEG time series, parallel LSTM is utilized. Parallel LSTM has 3 LSTM modules that have the same structure. Therefore, the three $Pt$ are converted by the parallel LSTM into 3 segments $L_{n1}$, $L_{n2}$, $L_{n3}$. $L_{n1}$,$L_{n2}$,$L_{n3}$ $\in R^{128}$, which are the final high-level representations of the EEG signals.

### Voting classifier

Based on the three high-level representations $L_{n1}$,$L_{n2}$,$L_{n3}$ of EEG signals, we apply three softmax activation functions, which can be defined as follows:

3 $$y_{1} = {\text{softmax}}\left( {A_{1} L_{n1} + B_{1} } \right)$$

4 $$y_{2} = {\text{softmax}}\left( {A_{2} L_{n2} + B_{2} } \right)$$

5 $$y_{3} = {\text{softmax}}\left( {A_{3} L_{n3} + B_{3} } \right)$$

$$A_{1} ,B_{1} ,A_{2} ,B_{2} ,A_{3} ,B_{3}$$ are learnable parameters, and $$y_{1} ,y_{2} ,y_{3} \in R^{3}$$ denotes the probability of $$y_{1} ,y_{2} ,y_{3}$$ belonging to all 3 classes. Specifically, the class of the largest probability is the predicted label $$Y_{1} ,Y_{2} ,Y_{3}$$. $${\mathrm{Y}}_{1},{\mathrm{Y}}_{2}{,\mathrm{Y}}_{3}\in$$$$Y_{1} ,Y_{2} ,Y_{3}$$$$\in$$[$$-$$ 1, 0, 1].

Due to the relative independence of the three outputs $L_{n}$ from the parallel LSTM, we use voting to aggregate the predicted labels for classification. In the parallel model, if the two prediction labels are consistent, the label is the final prediction of the model; if the predictions are inconsistent, the result ranked by time is the prediction. The final loss is the sum of MMD and CrossEntropyLoss.

## Experiment

In this section, we introduce 4 widely used datasets. Then, the experimental setting of our method is described. Finally, the results on the dataset are reported and discussed.

### SEED dataset

SEED is a public dataset for EEG emotion recognition collected by Shanghai Jiao Tong University and includes data from 15 subjects (7 males and 8 females) (Zheng and Lu 2015). The researchers selected 15 emotional clips from movies. Each clip is approximately 4 min long and contains only one kind of emotion. All film clips can be divided into three categories of emotions (positive, neutral and negative), which means five clips correspond to one emotion. Three groups of experiments were carried out for each subject. During the experiment, the EEG signals of the subjects were collected using a 62-channel ESI NeuroScan System with a sampling rate of 1000 Hz.

### SEED-IV dataset

The SEED-IV dataset (Zheng et al. 2019a, b) includes EEG emotion data from four categories: neutral, sad, fear, and happy. This dataset follows a similar structure as the SEED dataset and also involves 15 subjects. Three sessions of experiments were conducted for each subject, and each session contained 24 trials. Their EEG signals and eye movements were collected with the 62-channel ESI NeuroScan System and SMI eye-tracking glasses.

### DEAP dataset

The DEAP dataset provides a comprehensive collection of physiological data, comprising 32-channel EEG signals and self-reported emotional responses from 32 participants (Koelstra et al. 2011). During the experiment, participants were stimulated by 40 one-minute-long video clips. For each video clip, participants were requested to rate their level of valence and arousal on a scale ranging from 1 to 9. To simplify the label distribution, a threshold of 5 is employed to classify the labels into two classes. The EEG signals are recorded using a Biosemi ActiveTwo system, which including a 3-s baseline signal and a 60-s trial signal. In preprocessing, the DE features of baseline signal are subtracted from the DE features of the trial signal. This adjustment aims to eliminate the influence of the baseline EEG activity.

### DREAMER dataset

The DREAMER is a multimodal dataset designed for emotion recognition research (Katsigiannis and Ramzan 2017). It includes EEG, ECG data, and subjective emotion ratings obtained from 23 participants. Each participant was stimulated by 18 different audio and video clips. The EEG signals were recorded using a 14-channel Emotiv EPOC system and sampled at a rate of 128 Hz. Participants were asked to rate the videos based on their emotional experience. These labels are categorized into two classes using a threshold value of 3. For each video clip, baseline and trial EEG signals are collected. Similar to the DEAP dataset, the preprocessing involves subtracting baseline DE features from trial DE features.

### Experimental setup

The TDMNN was trained with a batch size of 64, and the maximum number of epochs was set as 150. Adam was used with a learning rate of 0.001. The model was implemented by Keras from Google TensorFlow and trained on an NVIDIA GeForce RTX 2080 Ti GPU. We used a protocol similar to that used by Shen et al. to evaluate the performance of EEG emotion recognition. Specifically, we performed a fivefold cross-validation and calculated the average classification accuracy (ACC) and the standard deviation (STD) for each subject.

### Baseline models

- DGCNN (Song et al. 2018): The DGCNN dynamically learns the intrinsic relationship between different EEG channels by an adjacency matrix and extracts advanced features from 5 initial features, such as the DE and PSD.
- BiHDM (Li et al. 2020): The BiHDM considers discrepancy information between the left and right hemispheres of the human brain and uses four RNNs to extract more discriminative features for EEG emotion recognition.
- RGNN (Zhong et al. 2022): The RGNN captures both local and global interchannel relations in EEG signals based on the EEG adjacency matrix.
- 4D-CRNN (Shen et al. 2020): The 4D-CRNN transforms the DE features into 4D structures and considers frequency, spatial and temporal information for emotion recognition.
- ACRNN (Tao et al. 2020): It uses a convolutional recurrent neural network to learn useful information in channel and time and introduces attention mechanisms to adaptively extract more discriminative features.
- MFBSE-EWT and ARF (Bhattacharyya et al. 2021): It is based on the application of the Fourier–Bessel series expansion and computes the spectral and temporal entropies from EEG signal for EEG emotion recognition.
- FBSE-EWT-based entropy features (Nalwaya et al. 2022): It decomposes the EEG using the FBSE-EWT into four modes and computes new FB-based entropy features, such as FB spectral-based SSE, LEE, and WE for emotion identification.
- FBSE-EWT and NCA feature selection with bagged tree (Anuragi et al. 2022): It considers wavelet entropy and energy features on different sub-band signals for capturing the temporal and spectral characteristics. The NCA and ensemble bagged tree classifiers are applied for emotion recognition.
- 4D-aNN (Xiao et al. 2022): Similar to our method, the 4D-aNN is also based on the structure of 4D-CRNN, and considers spatial, spectral, and temporal information of EEG signals. Different from our method, it uses attention mechanisms to capture critical information rather than temporal stability.
- V-IAG (Song et al. 2023): It simultaneously captures the individual dependencies among EEG channels and the underlying uncertain information using a variational instance-adaptive graph model.

## Results

Our model demonstrates the computational complexity of 31,919,659 FLOPs and 15,251,831 trainable parameters. And the prediction time is calculated for each dataset. For the SEED dataset, the average prediction time per subject amounts to 1487 s. Then, the average prediction time is 427 s on the DEAP dataset. Finally, for the DREAMER dataset, the average prediction time is 557 s.

To evaluate the performance of adopting the time stability of emotional activity, we compare the TDMNN with traditional methods on the SEED, SEED-IV, DEAP and DREAMER datasets. First, we conduct the experiment and the recognition accuracy of our method is compared with 10 baseline models. Then, we investigate the effect of EEG segment length (parameter *T*), which is an important hyperparameter, on the recognition accuracy. Finally, to identify that adopting time stability is helpful to improve the emotion recognition ability, an ablation experiment is conducted on the SEED dataset. We evaluate the performance of the TDMNN when the temporal-difference evaluation module and multibranch strategy are ablated. Our method achieves state-of-the-art performances and shows less sensitivity to the segment length parameter. The ablation experiment shows that removing the temporal-difference evaluation module and multibranch strategy have a certain impact on emotion recognition.

### Comparison of the accuracy of emotion recognition

**Fig. 3:**
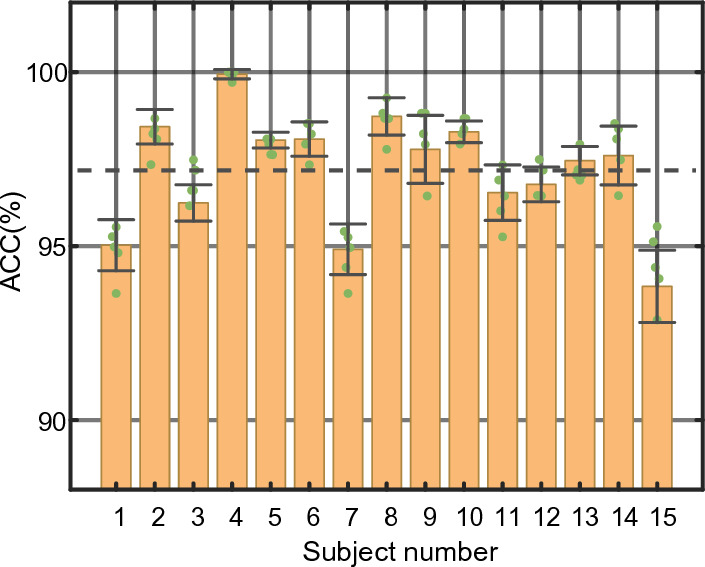
Performance of the TDMNN model on SEED dataset. The amount of scatter reflects the classification accuracy in fivefold cross-validation. The bar indicates the average ACC in fivefold cross-validation. The error bar indicates the standard deviation of the ACC. The dashed line is the average ACC for the 15 subjects

**Fig. 4:**
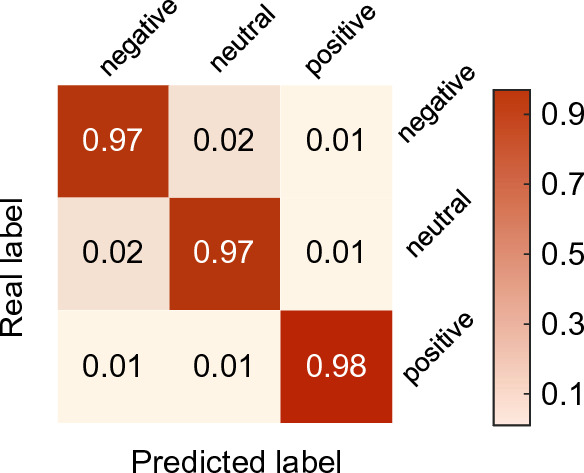
The confusion matrix of the TDMNN on SEED dataset. The element (i, j) is the percentage of samples in class i that are classified as class j

**Fig. 5:**
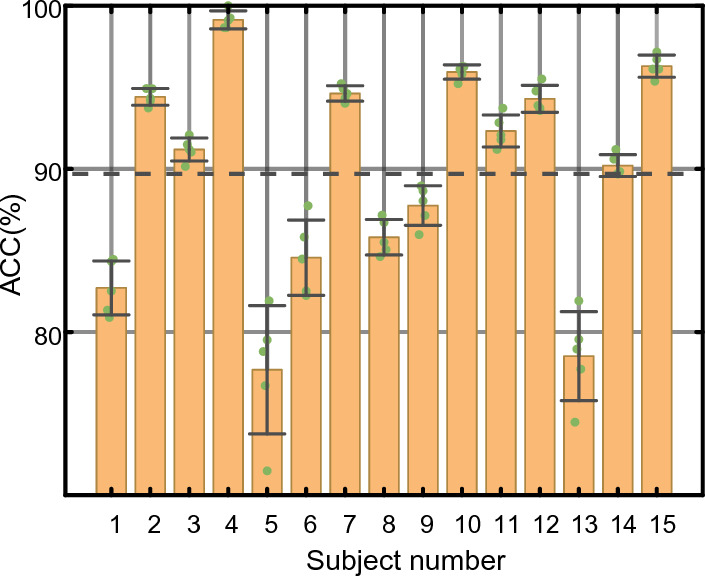
Performance of the TDMNN model on SEED-IV dataset

We evaluate the overall performance of our model, set the length of EEG segment *T* as 3, and carry out the experiment on SEED, SEED-IV, DEAP and DREAMER datasets. On the SEED dataset, the emotion classification accuracy is shown in Fig. 3. The accuracy of 15 subjects is above 90%, and the average ACC and STD of emotion classification are 97.20% and 1.57%, respectively. To evaluate the recognition ability of each emotion, we separately depict the average confusion matrix of all subjects in Fig. 4. It can be seen that for the SEED dataset, positive emotion is often easier to identify than neutral and negative emotions. The classification accuracy of positive, neutral and negative emotions reaches over 97%. On the SEED-IV dataset, the four-category average ACC and STD of TDMNN reaches 89.70% and 6.61% in Fig. 5. Among the 15 subjects, the average accuracy was exceeded for 9 subjects (#2, #3, #4, #7, #10, #11, #12, #14 and #15).

**Fig. 6:**
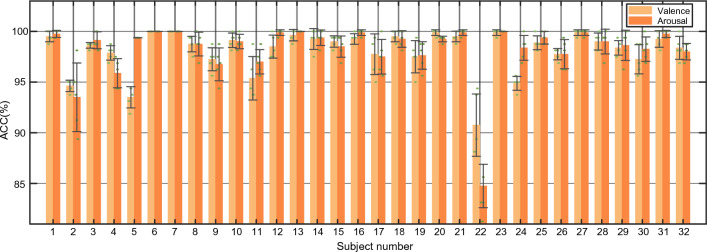
Performance of the TDMNN model on DEAP dataset. We conduct a fivefold cross-validation for each subject for valence and arousal classification

**Fig. 7:**
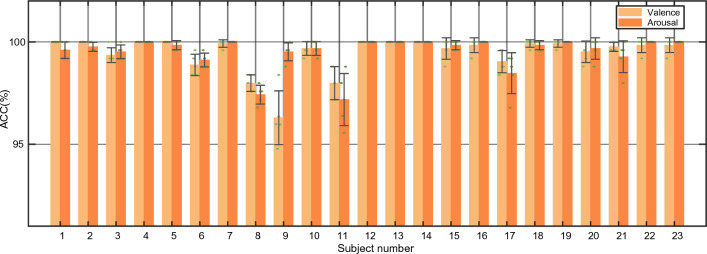
Performance of the TDMNN model on DREAMER dataset. We conduct a fivefold cross-validation for each subject for valence and arousal classification

Besides, we demonstrate the performances of TDMNN on the DEAP dataset and DREAMER dataset, as shown in Figs. 6 and 7. The DEAP dataset demonstrates impressive results in valence and arousal classification, with an average accuracy of 98.08% and 98.25% respectively. The majority of subjects achieve accuracy rates above 90%, with the exception of subject #22. Moving on to the DREAMER dataset, the average accuracy for valence and arousal classification is even higher at 99.45% and 99.51% respectively. Notably, all subjects in this dataset achieve accuracy above 95%.

Finally, we conduct leave-one-subject-cross-validation for subject-independent EEG emotion recognition on the SEED dataset. The accuracy of each subject is between 50 and 70%. Although the method achieved state-of-the-art performance within-participant, it still remains a challenge to improve the performance in cross-participant classification and needs further study in the future.

We compare our model with 10 baseline models. Table 1 presents the average ACC and STD of these models for EEG emotion recognition on SEED, SEED-IV, DEAP, and DREAMER dataset. On SEED dataset, the classification accuracy of TDMNN is 97.20%, beating DGCNN, BiHDM, RGNN, 4D-CRNN, “MFBSE-EWT and ARF”, “FBSE-EWT and NCA feature selection with bagged tree”, 4D-aNN, V-IAG by 6.80%, 4.08%, 2.96%, 2.46%, 2.80%, 1.50%, 0.95% and 1.56% respectively. For the SEED-IV dataset, the four-category classification accuracy of TDMNN reaches 89.70%, which outperforms 2.93% over 4D-aNN. For the DEAP dataset, the valence classification accuracy of TDMNN reached 98.08%, surpassing ACRNN, 4D-CRNN, and 4D-aNN by 4.36%, 3.86%, and 1.18% respectively. Furthermore, the arousal classification accuracy of TDMNN reached 98.25%, outperforming ACRNN, 4D-CRNN, and 4D-aNN by 4.87%, 3.67%, and 0.86% respectively. On DREAMER dataset, the valence and arousal classification accuracy of TDMNN reaches 99.45% and 99.51%, which are 1.52% and 1.53% higher than the highest accuracy achieved by ACRNN among the 10 baseline models.

**Table 1:** The performance (average ACC and STD (%)) of the compared models

Model	SEED	SEED-IV	DEAP-valence	DEAP-arousal	DREAMER-valence	DREAMER-arousal
DGCNN	90.40 $\pm$ 8.49	−	−	−	86.23 $\pm$ 12.3	84.54 $\pm$ 10.78
BiHDM	93.12 $\pm$ 6.06	74.35 $\pm$ 14.09	−	−	−	−
RGNN	94.24 $\pm$ 5.95	79.37 $\pm$ 10.54	−	−	−	−
4D-CRNN	94.74 $\pm$ 2.32	−	94.22 $\pm$ 2.61	94.58 $\pm$ 3.69	−	−
ACRNN	−	−	93.72 $\pm$ 3.21	93.38 $\pm$ 3.73	97.93 $\pm$ 1.73	97.98 $\pm$ 1.92
MFBSE-EWT and ARF	94.40	−	−	−	86.20	85.40
FBSE-EWT-based entropy features	−	−	−	−	97.91	97.84
FBSE-EWT and NCA feature selection with bagged tree	95.70	−	83.90	84.30	−	−
4D-aNN	96.25 $\pm$ 1.86	86.77 $\pm$ 7.29	96.90 $\pm$ 1.65	97.39 $\pm$ 1.75	−	−
V-IAG	95.64 $\pm$ 5.08	−	−	−	92.82	93.09
**TDMNN (ours)**	**97.20 **$\pm$** 1.57**	**89.70** $\pm$ **6.61**	**98.08 **$\pm$** 2.13**	**98.25 **$\pm$** 2.85**	**99.45 **$\pm$** 0.91**	**99.51 **$\pm$** 0.79**

The bold denotes the best performance

### EEG segment length effect

**Fig. 8:**
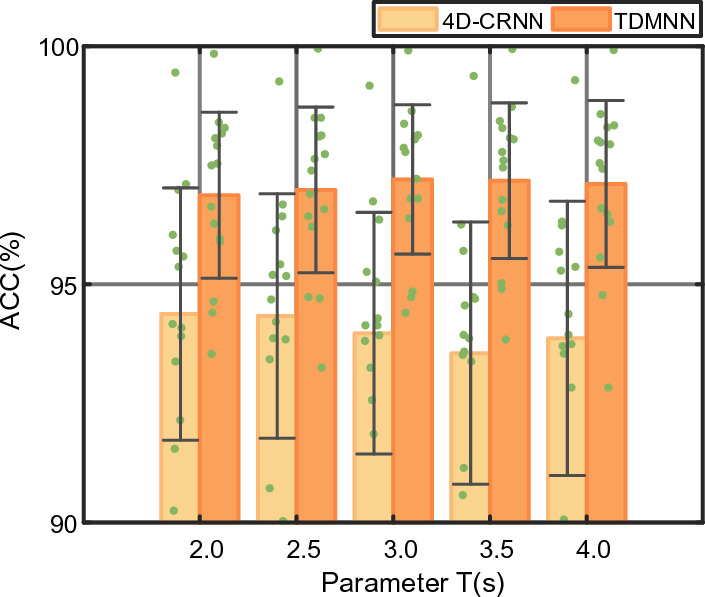
The performance of emotion classification on SEED dataset in different lengths for the 4D-CRNN (lite yellow) and TDMNN (deep yellow) models. The amount of scatter reflects the classification accuracy for the 15 subjects. The bar indicates the average ACC for the 15 subjects. The error bar indicates the standard deviation of the ACC for the 15 subjects

The spatial and temporal features contained in each EEG segment rely on the parameter *T* (the length of EEG segments). Therefore, we study the influence of different lengths of EEG segment on the performance of emotion classification based on SEED dataset, as shown in Fig. 8. Because the 4D-CRNN, which lacks the temporal-difference evaluation module and multibranch strategy, is the basis of the TDMNN, we compare the sensitivity to *T* with the 4D-CRNN.

We investigate the segment length *T* with ranges [2, 2.5, 3, 3.5, 4] on the SEED dataset. The average accuracies of the TDMNN are 96.87%, 96.98%, 97.20%, 97.18%, and 97.11%, respectively. The results show that the accuracy of emotion recognition based on the TDMNN is generally higher than that based on the 4D-CRNN. Moreover, the standard deviation in different $T$ is 0.34% for the 4D-CRNN and 0.14% for the TDMNN. The result shows that the performance of the TDMNN is more stable across different *T*. From the above, it can be seen that our method is less sensitive to the parameter *T*, implying that the TDMNN is not easily affected by inadequate parameters.

**Fig. 9:**
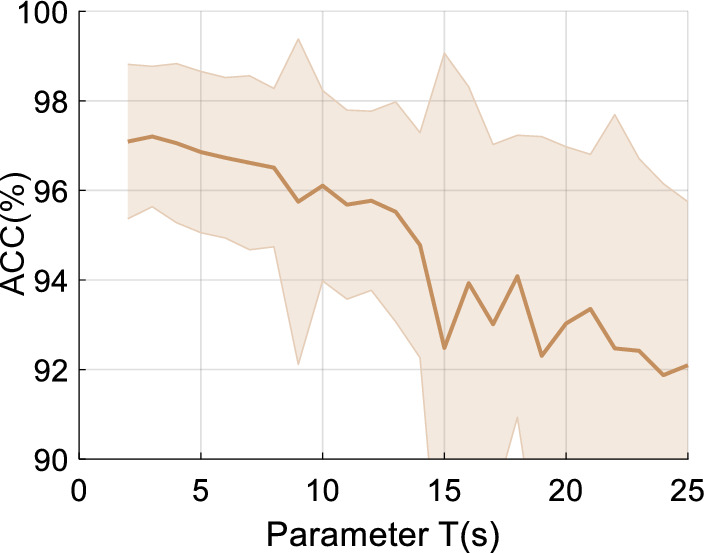
The accuracy of TDMNN versus length of EEG segment (parameter *T*)

Furthermore, in order to explore the influence of length of EEG segment(period) on the performance of our method, we study the accuracy of TDMNN in different lengths of EEG segment (parameter *T*), as shown in Fig. 9. It can be seen that the accuracy of our model is relatively high when *T* is smaller than 12. And the highest accuracy is achieved when *T* is 3. Thus, we set the parameter *T* as 3 in our work.

### Ablation experiments

To study the effect of adopting the time stability of emotional activity, we design an ablation experiment, in which we compare the performance of the TDMNN based on SEED dataset when the temporal-difference evaluation module, multibranch strategy and both are ablated, as depicted in Table 2.

**Table 2:** Comparison of the ablation experiment on SEED dataset. “−” denotes the ablation of certain modules

Model	ACC $\pm$ STD (%)	P (%)	R (%)	Macro-F1 (%)
TDMNN	97.20 $\pm$ 1.57	97.73	97.72	97.71
$-$ Temporal-difference evaluation module	96.47 $\pm$ 2.00	97.06	97.05	97.04
$-$Multibranch strategy	95.54 $\pm$ 2.23	96.09	96.06	96.06
$-$Both modules	93.97 $\pm$ 2.54	94.20	94.02	94.02

First, we evaluate the performance of the TDMNN when the temporal-difference evaluation module is ablated. The 2* T* feature vectors from the CNN are randomly arranged into 3 orders for the prediction of the voting classifier without the temporal-difference evaluation module. The length of the EEG segment (*T*) is set as 3. Fivefold cross-validation is performed on each subject. The average ACC $\pm$ STD of emotion classification is 96.47 $\pm$ 2.00%. Then, we evaluate the performance of the TDMNN when the multibranch strategy is ablated. The average ACC $\pm$ STD of emotion classification is 95.54 $\pm$ 2.23%. Finally, we evaluate the performance of the TDMNN when both the temporal-difference evaluation module and multibranch strategy are ablated. We set the same input as before, and only 93.97 $\pm$ 2.54% is achieved.

From the ablation experiment, we find that removing the temporal-difference evaluation module and multibranch strategy could impact the performance compared with the baseline model, and the average accuracy decreases by 0.73% and 1.66%, respectively. Specifically, the accuracy of the model after removing both the module and strategy decreases by 3.23%. We also analyse the precision (P), recall (R) and macro-F1 score and find that they are all affected, as shown in Table 2. The average P, R and macro-F1 score of the TDMNN are 97.73%, 97.72% and 97.71%, respectively. When both methods are removed from the TDMNN, the average P, R and macro-F1 score decrease by 3.53%, 3.70% and 3.69%, respectively.

## Discussion

We conduct several experiments to analyse whether involving prior knowledge of the time stability of emotional activity is helpful for achieving efficient emotion recognition. In this section, five noteworthy points will be discussed.

First, emotional activities are relatively stable, which leads to similarities in neural patterns over time (Zheng et al. 2019a, b). In the field of EEG-based emotion recognition, researchers ignored the temporal relationship between different segments and used different EEG segments separately. It is still challenging to make efficient use of the stability of emotional activities. Based on prior knowledge, we propose a TDMNN for EEG emotion recognition using temporal stability. The average accuracy of emotion recognition by our method is much higher than that of the baseline models. It has been verified by our experiment that stability improves the performance of emotion recognition.

Second, in this work, the temporal stability of emotional activity is involved in the proposed temporal-difference evaluation module and multibranch strategy. The temporal-difference evaluation module aims to evaluate the differences in features at different times (sections). We attempted to add the evaluated difference value to the loss function to guide the training of the neural network. In this work, the MMD tool is used to evaluate the temporal-difference value. However, the MMD tool can only evaluate the difference between two groups, not among several sections. To efficiently evaluate the temporal difference in EEG time series, a multibranch strategy is adopted. In each branch, 6 sections among 3 s are randomly divided into 2 groups, and the difference between the 2 groups is evaluated separately. Therefore, the TDMNN can make full use of the difference among the sections to obtain the time stability of emotional activities. The proposed method achieves state-of-the-art performance on benchmark datasets. In addition, we conduct ablation studies on different modules. The experimental results demonstrate the effectiveness of the proposed temporal-difference evaluation module and multibranch strategy to evaluate the temporal stability of emotional activity.

Third, the stability of emotional activities is only effective for a certain length of time, so the division of the EEG segment (parameter *T*) is an important parameter. When the parameter *T* is small, the time stability is strong within a segment. However, a shorter EEG segment carries less emotional information. Setting an appropriate parameter may be important for the performance of the emotion recognition method (Ouyang et al. 2022). If the algorithm is sensitive, it is difficult to select the appropriate parameter *T*. The sensitivity of the emotion recognition method to the EEG segment should be considered. For our method, the accuracy of emotion recognition changes less when parameter *T* changes. This makes it easy to determine a proper parameter *T* for satisfactory emotion recognition performance.

Fourth, in our work, the multichannel EEG signals have been used for extracting more abundant EEG features. We decomposed the EEG signal into four frequency bands, and the DE features are calculated on each band. It was reported that the frequency features play an important role in EEG emotion recognition (Shen et al. 2020). Therefore, the frequency decomposition method may be important. Recently, many multichannel decomposition techniques have been proposed. Bhattacharyya and Pachori explored the empirical wavelet transform (EWT) for the multivariate signals (Bhattacharyya and Pachori 2017). Nalwaya et al. introduced FBSE-based empirical wavelet transform (FBSE-EWT) to decompose the EEG signals into narrow-band modes (Nalwaya et al. 2022). Introducing these new multichannel decomposition techniques may increase the performance of our emotion recognition method and this needs further study.

Fifth, we only showed the result using DE features in this paper. Other sets of features, such as power spectral density (PSD) and Fourier–Bessel domain differential entropies (FBDE), have been tried for feature extraction in our method. PSD provides a way of representing the distribution of signal frequency components, which have been proven to be effective for emotion recognition (Zheng and Lu 2015). FBDE is the differential entropy obtained from the signal after using the Fourier–Bessel series expansion (FBSE) (Nalwaya et al. 2022). The results showed that the performances on DE, PSD, and FBDE were very close and all achieved an accuracy rate of approximately 98%. The accuracy on DE was slightly higher than others. Thus, the results only using DE features are given in the work.

## Conclusion

In this paper, we propose the TDMNN model, which considers the knowledge that emotional activities vary slowly over time. By using the TDMNN model, more discriminative features are further extracted. The vital procedures lie in that the maximum mean discrepancy technology is utilized to evaluate the difference of features across time, and a multibranch convolutional recurrent network is used to minimize the difference. The proposed method achieves state-of-the-art performance on the SEED, SEED-IV, DEAP and DREAMER datasets and shows less sensitivity to the proper parameter $T$ for satisfactory emotion recognition performance. The experiments verify that involving the prior knowledge of time stability of the emotional activity has a certain improvement on emotion recognition.

## Data Availability

Codes are available at https://github.com/jxygithub123/TDMNN.
